# Adequacy of compression positioning using the feedback device during chest compressions by medical staff in a simulation study

**DOI:** 10.1186/s12873-022-00640-6

**Published:** 2022-05-06

**Authors:** Yasuaki Koyama, Tasuku Matsuyama, Takako Kaino, Tetsuya Hoshino, Junzo Nakao, Nobutake Shimojo, Yoshiaki Inoue

**Affiliations:** 1 Department of Emergency and Critical Care Medicine, University of Tsukuba Hospital, 2-1-1 Amakubo, Tsukuba, Ibaraki Japan; 2 Department of Emergency Medicine, Kyoto Prefectural University of Medicine, 465 Kawaramachi-Hirokoji, Kamigyo-ku, Kyoto Japan

**Keywords:** Chest compressions, Feedback device, Adequate position for chest compressions, Flexible pressure sensor, Hypothenar side

## Abstract

**Background:**

The 2020 American Heart Association guidelines recommend the use of a feedback device during chest compressions (CCs). However, these devices are only placed visually by medical personnel on the lower half of the sternum and do not provide feedback on the adequacy of the pressure-delivery position. In this study, we investigated whether medical staff could deliver CCs at the adequate compression position using a feedback device and identified where the inadequate position was compressed.

**Methods:**

This simulation-based, prospective single-centre study enrolled 44 medical personnel who were assigned to four different groups based on the standing position and the hand in contact with the feedback device as follows: right–*left* (R–*l*), right–*right* (R–*r*), left–*right* (L–*r*), and left–*left* (L–*l*), respectively. The sensor position where the maximal average pressure was applied during CCs using the feedback device were ascertained with a flexible capacitive pressure sensor. We determined if this position is the adequate compression position or not. The intergroup differences in the frequency of the adequate compression position, the maximal average pressure, compression rate, depth and recoil were determined.

**Results:**

The frequencies of adequate compression positioning were 55, 50, 58, and 60% in the R–*l*, R–*r*, L–*r,* and L–*l* groups, respectively, with no significant intergroup difference (*p* = 0.917). Inadequate position occurred in the front, back, hypothenar and thenar sides. The maximal average pressure did not significantly differ among the groups (*p* = 0.0781). The average compression rate was 100–110 compressions/min in each group, the average depth was 5–6 cm, and the average recoil was 0.1 cm, with no significant intergroup differences (*p* = 0.0882, 0.9653, and 0.2757, respectively).

**Conclusions:**

We found that only approximately half of the medical staff could deliver CCs using the feedback device at an adequate compression position and the inadequate position occurred in all sides. Resuscitation courses should be designed to educate trainees about the proper placement during CCs using a feedback device while also evaluating the correct compression position.

## Background

The 2020 International Consensus guidelines recommend the use of a feedback device for cardiopulmonary resuscitation (CPR) training [1] to facilitate effective CPR based on the evaluation of its components, including compression depth, compression rate, chest wall recoil, and interruption time [2–6]. Some clinical studies demonstrated that CPR depth and rate were significantly estimated better with a feedback device [7]. The return of spontaneous circulation (ROSC) and survival discharge rates were also higher with it [8].

However, the feedback device is only placed visually by medical personnel on the lower half of the sternum. In general, feedback devices cannot determine whether they are in the appropriate place and in the adequate pressure position. Proper device placement is important because CCs in the inadequate position results in several complications, including sternal and rib fractures. The number of sternal fractures was slightly smaller when the compression position was on the lower half of the sternum than in the internipple line [9–11]. Meanwhile, the fourth rib is the most common site for rib fractures, and these incidences can be improved by correcting the compression position during manual CCs performed by medical staff [12, 13]. Therefore, it is necessary to investigate the adequate position of CCs using feedback devices, based on objective assessment. In this study, we evaluated our hypothesis that medical staff can deliver CCs using feedback devices at the adequate position.

## Methods

### Study design and participants

In this simulation-based, prospective, single-centre study, 10 doctors and 20 nurses from our hospital, and 14 paramedics were enrolled. The doctors were the residents or staff in the department of emergency and critical care medicine, and the nurses worked in the emergency department or intensive care. The paramedics had national Emergency Life-saving Technician certification and were trained in resuscitation within each fire station. The study protocol was approved by the institutional ethics committee of the University of Tsukuba Hospital (H30–355). All participants gave written informed consent for study participation.

### Materials for practice

#### Study instruments

**Fig. 1:**
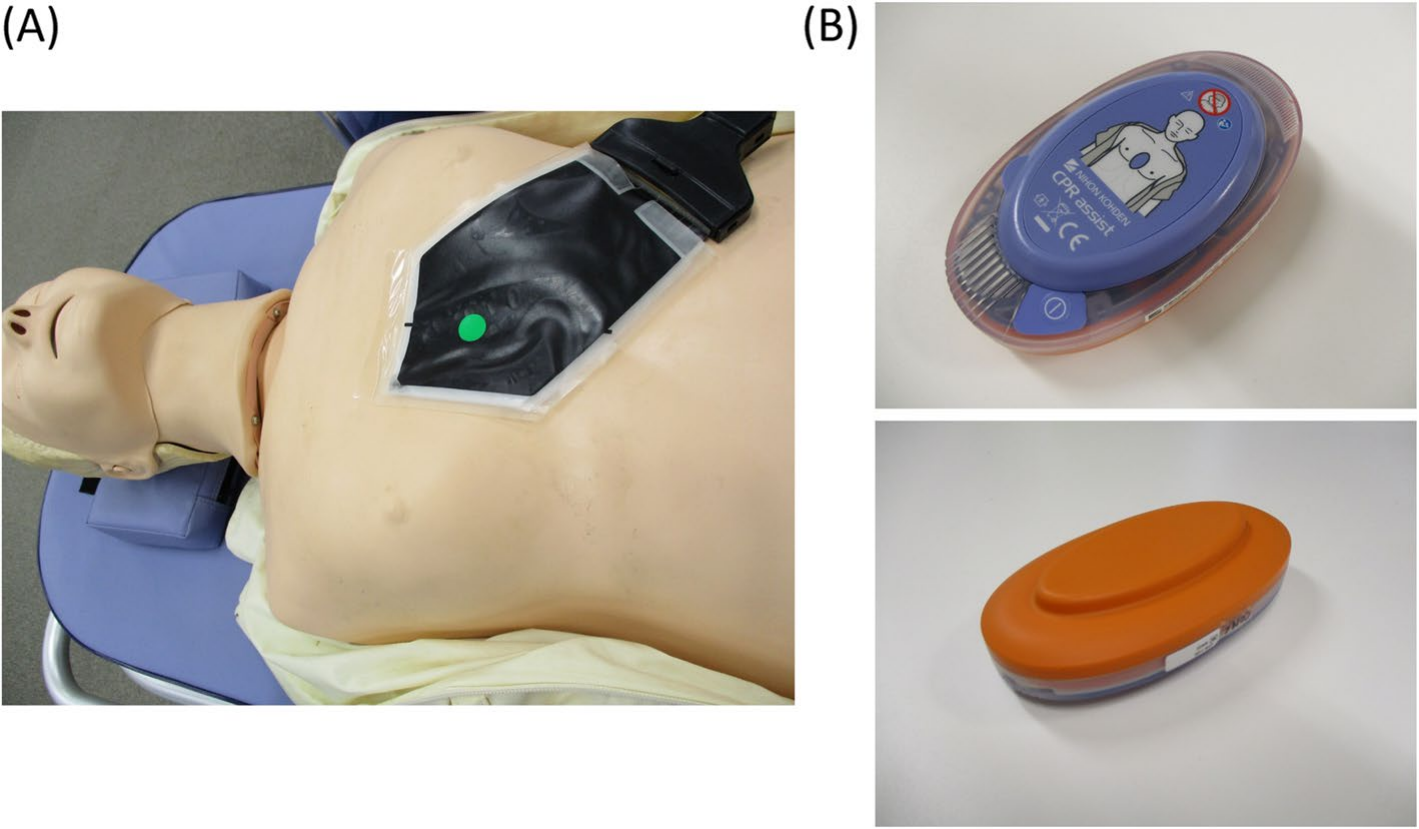
The pressure sensor and feedback device. **A** The flexible pressure sensor (Shinnosuke-kun™) was situated on the lower half of the sternum in the Little Anne™ CPR training manikin. The adequate position was assigned as a 3 × 3 cm^2^ area centered on the green dot. **B** The feedback device (CPR-Assist™) with the ventral view (Top) and dorsal view (Bottom)

A flexible capacitive pressure sensor (Shinnosuke-kun™, Sumitomo Riko Corporation, Komaki-shi, Japan) was used to measure the exact position and pressure (expressed in picofarad) during CCs. The manikin (Little Anne™ CPR Training Manikin, Laerdal Medical Corporation, Stavanger, Norway) was placed on a stretcher, and the flexible sensor was fixed with double-sided tape after positioning its central mark on the lower half of the manikin’s sternum and verifying accurate placement (Fig. 1a). The flexible sensor (total sensor size: 5 × 5 cm^2^) included 25 pressure sensors (one sensor size: 1 × 1 cm^2^). We assigned the adequate position as 3 × 3 cm^2^ area, which was the centre of the flexible sensor as previously reported [14].

A feedback device (CPR-Assist™, Nihon Kohden Corporation, Tokyo, Japan) was used (Fig. 1b). The areas for hand placement and sternal contact were 9 × 6 cm^2^ and 9 × 4 cm^2^, respectively, and the thickness was 3 cm. The length of the lower half of the adult sternum is approximately 10 cm [15]. Therefore, the CPR-Assist™ was placed on the lower half of the sternum, so the centre of the feedback device was placed at the central mark. No feedback from the manikin, the flexible sensor, and the feedback device was provided to the medical staff participants during the CC procedure.

#### Study procedure

The study participants were randomly and evenly divided within their professional groups into two order protocols based on the CC method - each order protocol had five doctors, 10 nurses, and seven paramedics. The participants placed the CPR-Assist™ on the central mark and selected the same hand to contact the feedback device, that was in contact with the sternum during the manual CCs.

According to the first order protocol, the CPR-Assist™ was placed from the right side at first and CCs were performed for 1 min, followed by at least a 1-min interval before placing the CPR-Assist™ from the left side to perform CCs for another 1 min (Order rt). Similarly, for the other order protocol, CCs were performed at first from the left side for 1 min, with an interval of at least 1 min, before performing CCs from the right side for 1 min (Order lt).

#### Data collection

Participant data, including age, sex, profession, work experience, dominant hand, CPR training and CPR experience within 2 years of the study, and CPR-Assist™ experience, were collected (Table 1).

**Table 1:** Characteristics of the participants in the two order protocols

Characteristics	Order rt	Order lt	***p***-value
Participants, *n*	22	22	
Ages (years), mean ± SD	31 ± 7	33 ± 7	0.5064
Sex, *n*			0.5394
Male	14	16	
Female	8	6	
Profession, *n*			1
Doctor	5	5	
Nurse	10	10	
Ambulance crew	7	7	
Work experience (years), mean ± SD	7.5 ± 5.3	8.2 ± 6.1	0.5332
Dominant hand, *n*			1
Right	21	20	
Left	1	2	
CPR training within 2 years, *n*			0.2027
Yes	17	12	
No	5	10	
CPR experience within 2 years, *n*			0.4121
Yes	20	17	
No	2	5	
CPR-Assist experience, *n*			0.6069
Yes	3	1	
No	19	21	

Order rt.: CPR-Assist™ was placed from the right side at first and CCs were performed for 1 min, followed by at least a 1-min interval before placing the CPR-Assist™ from the left side to perform CCs for another 1 min. Order lt: CPR-Assist™ was placed from the left side at first and CCs were performed for 1 min, with an interval of at least 1 min, before placing the CPR-Assist™ from the right side to perform CCs for another 1 min

*CC* chest compression, *CPR* cardiopulmonary resuscitation, *SD* standard deviation

**Fig. 2:**
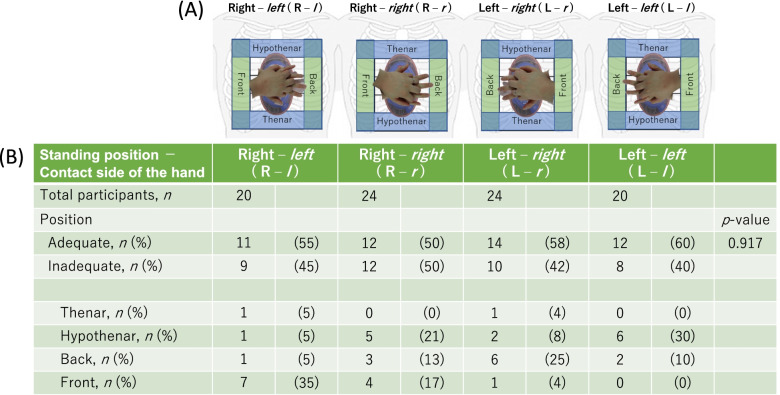
The frequency of adequate and inadequate compression positioning. **A** The figures are in the standing position, hand in contact with the CPR-Assist™, and definition of inadequate position. The front label indicates the base side of the hand in contact with the CPR-Assist™, whereas the back label indicates the tip side of the finger; the thenar and hypothenar label indicate the thenar and hypothenar side of the hand, respectively. **B** The inset table shows the primary outcomes, namely the frequency of adequate compression positioning where the maximal average pressure was applied to the sensor for each participant, the location where the inadequate position occurred in, and the frequency of each inadequate position

We examined the sensor position where the maximal average pressure was applied to all the 25 sensors by each participant. The collected data were divided into four groups according to the standing position and the freely selected hand during CPR-Assist™ contact. When the participants performed CCs from the right side: the right–*left* (R–*l*) group placed the left hand and the right–*right* (R–*r*) group placed the right hand in contact with the CPR-Assist™. Similarly, when the participants performed CCs from the left side: the left–*right* (L–*r*) group placed the right hand and the left–*left* (L–*l*) group placed the left hand in contact with the CPR-Assist™ (Fig. 2a).

### Outcome measures

The primary outcomes included the ratio of the adequate compression position where maximal average pressure was applied to the sensors by each participant, to ascertain the adequacy of compression position, the location to where the inadequate position occurred (the front, back, hypothenar and thenar sides), and the frequency of each inadequate position. The secondary outcomes included the maximal average pressure applied to each sensor, the average depth, the average compression rate, and the average recoil for each participant. The length of the recoil was measured by the depth of the part of the rib cage that had not returned, which was recorded as 0 cm if the rib cage had completely returned.

### Statistical analysis

Background and clinical data are presented as median (interquartile range) or proportions. The chi-square and Kruskal–Wallis H tests were used for categorical and continuous variables, respectively. We used the Mann–Whitney *U*-test with Bonferroni correction if there was a significant difference. Intergroup comparisons were conducted based on the standing position and hand in contact with the CPR-Assist™, and the four methods were compared. All *p*-values were two-sided, and *p* ≤ 0.05 was considered as statistically significant. All statistical analyses were conducted using EZR (Saitama Medical Centre, Jichi Medical University, Saitama, Japan) [16], a graphical user interface for R (The R Foundation for Statistical Computing, Vienna, Austria), which is a modified version of the R Commander designed to include statistical functions that are frequently used in biostatistics.

## Results

### Patient characteristics

This study included 30 men and 14 women. Four of the 44 total participants had prior CPR-Assist™ experience. The average age and work experience of the participants were 32 ± 7 years and 7.8 ± 5.7 years, respectively. There were no significant differences between the two order protocols (Table 1).

### Adequate compression position with CPR-assist™

There were 20, 24, 24, and 20 participants in the R–*l*, R–*r*, L–*r*, and L–*l* groups, respectively (Fig. 2). The proportions of the adequate compression position were 55, 50, 58, and 60% in the R–*l*, R–*r*, L–*r,* and L–*l* groups, respectively, without significant intergroup differences (*p* = 0.917). The inadequate position occurred in all sides (Fig. 2), but predominantly occurred by 35% in the front side in the R–*l* group, 21% in the hypothenar side in the R–*r* group, 25% in the back side in the L–*r* group, and 30% in the hypothenar side in the L–*l* group.

### Secondary outcomes

**Fig. 3:**
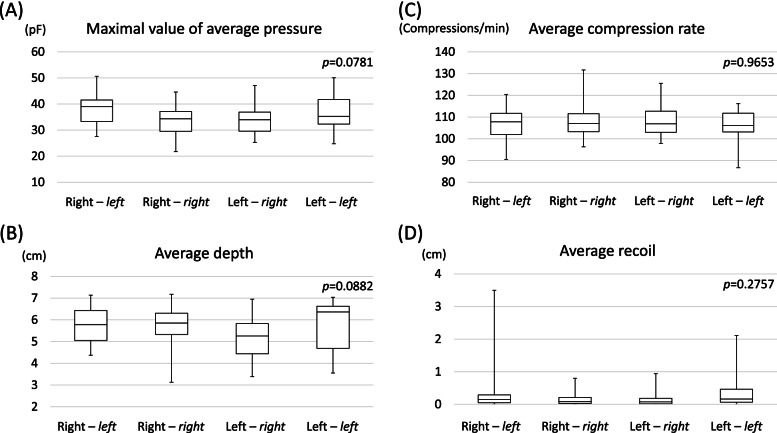
The secondary outcomes that included the maximum value of the average pressure applied to each sensor (**A**), the average depth (**B**), the average compression rate (**C**), and the average recoil (**D**) in all groups

There was no significant intergroup difference (*p* = 0.0781) in the maximal average pressure applied to each sensor (Fig. 3a). For the R–*l*, R–*r*, L–*r*, and L–*l* groups, the median average CC depths were 5.78, 5.85, 5.26, and 6.36 cm (Fig. 3b), while the average compression rates were 107.8, 107.0, 106.9, and 106.1 compressions/min (Fig. 3c) for the R–*l*, R–*r*, L–*r*, and L–*l* groups, respectively. Finally, the median average recoil were 0.15, 0.09, 0.07, and 0.16 cm for the R–*l*, R–*r*, L–*r*, and L–*l* groups, respectively (Fig. 3d). There were no significant intergroup differences in the average compression rate, average depth, and average recoil (*p* = 0.0882, 0.9653, and 0.2757, respectively).

## Discussion

In our study, only approximately 56% (min 50% - max 60%) of the medical staff could deliver CCs using the feedback device (CPR-Assist™) at the adequate compression position at the lower half of the sternum. Meanwhile, the inadequate position occurred in all sides.

In other studies that also used a flexible capacitive sensor [14, 17], only approximately 30% of the medical staff or ambulance crew could deliver manual CCs at the adequate position without a feedback device. Most of the rescuers pushed the sternum with the hypothenar side of their wrist joints when the hand was in dorsiflexion [14, 15, 18, 19]. Since the rescuers deliver manual CCs on soft tissues, such as skin and subcutaneous tissues in humans, and on soft materials, such as silicone, when simulating the human skin texture on manikins, it is likely that rescuers can only push with the hypothenar side of their hand at pinpoint. In contrast, when using a feedback device, such as the CPR-Assist™, the surface that is in contact with the lower hand is hard. Therefore, the rescuers push with both the thenar and hypothenar sides to disperse the force as much as possible. Therefore, the rescuers pushed the human patient or manikin with a narrower area of the hand than the total contact area of the CPR-Assist™ during CCs, which might have caused the inadequate compression position more clearly. This could be why the ratio of adequate compression positions using the feedback device in this study was higher than that in the manual CCs reported in the previous study [14].

Previous studies have reported that more than 90% of CCs using feedback devices were delivered in the correct position [2–5]. However, these studies used the Resusci Anne Skill Reporter Manikin™ (Laerdal Medical Corporation, Stavanger, Norway), which has a 10 × 10 cm^2^ plate on the back of the chest, or the Ambu®Man Torso (Ambu A/S, Ballerup, Denmark) CPR manikin, which has a 9 × 6 cm^2^ plate on the back of the chest. These devices have wider right and left margins than the Shinnosuke-kun™ with an adequate position of 3 × 3 cm^2^ area, that was used in this study. Therefore, the proportion of participants pushing at the correct position in other studies was higher than that in our study.

In this study, we found that the inadequate compression position occurred in the hypothenar side in the R–*r* and L–*l* subgroups, which poses a risk of pushing on the xiphoid that increases the risk of abdominal trauma, such as liver injury [20]. This result was similarly achieved by manual CCs [14] and the participants might have used the feedback device to deliver CCs resulting in the same sensation to manual CCs. Furthermore, if the plane compressed the thenar and hypothenar sides, the ligaments in between would be subjected to force, which could cause damage [18, 19]. The inadequate position also occurred in the front or back side in the R–*l* and L–*r* subgroups. This might be due to the shorter width of the right and left CPR-Assist™ relative to the length of the top and bottom; thus, a slight displacement could easily tilt the plane. The average width of the right and left sternal costochondral junction is 7.9 cm [21], and the width in contact with the sternum in the CPR-Assist™ is 4 cm. If the CPR-Assist™ and the compression force are displaced, force may be applied to the sternal costochondral junction, which increases the risk of fracture [9, 22]. As recommended in the guidelines [23], the feedback device must be placed in the median sternal region with special care.

The pressure applied to each sensor showed no significant intergroup differences. The compression depth and rate, which are important factors for the quality of CCs, were almost in accordance with the guideline recommendations (5–6 cm and 100–120 compressions/min, respectively) [23]. Most of the participants had never used a feedback device in resuscitation courses or in clinical settings. These participants might not have been able to obtain full chest recoil in this study due to the thickness of the feedback device, unlike the usual sensation during manual CPR situations without a feedback device.

One component of optimal CCs is the correct position, as recommended in the 2020 International Consensus guidelines [23]. In this study, only approximately half of the medical staff could deliver CCs using the feedback device at the correct placement, and although this proportion is better than manual CCs, it needs to be improved. Therefore, it is necessary to offer trainings on CCs using feedback devices in resuscitation courses, as well as the confirmation that the trainees can push at the adequate compression position.

### Limitations

There are some limitations of our study. First, each participant performed CCs for 1 min with intervals of at least 1 min to account for fatigue. In this study, CCs were not performed continuously every 2 min, as recommended in the guidelines. If CCs were performed similar to the recommended time, the depth and rate may have decreased with fatigue or damage, even though the placement of the feedback device was the same. Second, this study was conducted in a simulated setting, and it is still unknown if the use of feedback devices improves the positioning of CCs in actual clinical settings. Therefore, research on the delivery of CCs using feedback devices at the adequate compression position in clinical settings and how the quality of CCs worsens with fatigue while using these devices for 2 min are required.

## Conclusions

Only approximately half of the medical staff could deliver CCs using the feedback device at the adequate position over the lower half of the sternum. We also found that the inadequate compression position occurred in all sides. CPR instructors should educate trainees about the proper placement of CCs using feedback devices while evaluating the compression position during CPR courses.

## Data Availability

The data that support the findings of this study are available from the corresponding author, YK, upon reasonable request. The institutional ethics committee was approved that the data are not publicly and openly available due to human data.

## Abbreviations

CC: Chest compressions
CPR: Cardiopulmonary resuscitation
ROSC: Return of spontaneous circulation
